# Quantitative Methods to Detect Suicide and Self-Harm Clusters: A Systematic Review

**DOI:** 10.3390/ijerph19095313

**Published:** 2022-04-27

**Authors:** Ruth Benson, Jan Rigby, Christopher Brunsdon, Grace Cully, Lay San Too, Ella Arensman

**Affiliations:** 1 School of Public Health, College of Medicine and Health, University College Cork, Western Gateway Building, T12 XF62 Cork, Ireland; grace.cully@ucc.ie (G.C.); ella.arensman@ucc.ie (E.A.); 2 National Suicide Research Foundation, University College Cork, 4.28 Western Gateway Building, T12 XF62 Cork, Ireland; 3 National Centre for Geocomputation, Maynooth University, W23 F2H6 Maynooth, Ireland; jan.rigby@mu.ie (J.R.); christopher.brunsdon@mu.ie (C.B.); 4 Centre for Mental Health, Melbourne School of Population and Global Health, The University of Melbourne, Melbourne, VIC 3053, Australia; tiffany.too@unimelb.edu.au; 5 Australian Institute for Suicide Research and Prevention, School of Applied Psychology, Griffith University, Brisbane, QLD 4122, Australia

**Keywords:** systematic review, suicide, self-harm, cluster detection, contagion, geospatial analysis

## Abstract

Suicide and self-harm clusters exist in various forms, including point, mass, and echo clusters. The early identification of clusters is important to mitigate contagion and allocate timely interventions. A systematic review was conducted to synthesize existing evidence of quantitative analyses of suicide and self-harm clusters. Electronic databases including Medline, Embase, Web of Science, and Scopus were searched from date of inception to December 2020 for studies that statistically analyzed the presence of suicide or self-harm clusters. Extracted data were narratively synthesized due to heterogeneity among the statistical methods applied. Of 7268 identified studies, 79 were eligible for narrative synthesis. Most studies quantitatively verified the presence of suicide and self-harm clusters based on the scale of the data and type of cluster. A Poisson-based scan statistical model was found to be effective in accurately detecting point and echo clusters. Mass clusters are typically detected by a time-series regression model, although limitations exist. Recently, the statistical analysis of suicide and self-harm clusters has progressed due to advances in quantitative methods and geospatial analytical techniques, most notably spatial scanning software. The application of such techniques to real-time surveillance data could effectively detect emerging clusters and provide timely intervention.

## 1. Introduction

Suicide clusters are commonly referred to as a higher number of suicide deaths, attempted suicides, or self-harm events that occur in a population, location, or period than usually expected, based on statistical probability or community expectancy [1]; however, due to a lack of consensus regarding an operational definition of suicide clusters, particularly relating to the minimal number of cases that constitute a cluster, definitions are typically determined on an ad hoc basis in terms of the spatial and temporal limits of a cluster [2]. Clusters are suggested to be a product of a phenomenon known as contagion, whereby direct or indirect exposure to suicide results in subsequent suicide cases [3]. Suicide clusters are mostly reported within the adolescent population, particularly 15–24-year-olds [4,5] and are estimated to account for between 1% and 5% of all adolescent deaths by suicide [6,7]. Previous evidence suggests that an increase in the incidence of suicide clusters in recent years is linked to the broadening of social connections through electronic communication systems and internet-based social sites [8], particularly in the form of suicide pacts [9]. Geographical remoteness, economic deprivation, and indigenous status are factors associated with suicide clusters [10,11,12]. Furthermore, suicide clusters are more likely to occur in areas inhabited by disadvantaged cohorts, as certain risk factors associated with suicide including unemployment, socio-economic deprivation, and substance abuse occur more often in this population [13].

The patterns of suicide and self-harm mainly researched and documented within the literature are of two main types: mass clusters and point clusters. Mass clusters (or temporal clusters) involve a temporary increase in the total number of suicides within a population relative to the period before and after the cluster, with a lack of spatial relevance typically observed in the aftermath of a real or fictional suicide documented in the media [9,10]. In contrast, point clusters (or spatiotemporal clusters) are those that occur close together in both space and time within a given community or institution, and clusters of this nature can occur without the presence of media coverage [11]. A third pattern of suicide (spatial clusters) has been identified in the literature [2,3,4,5,6,7,8,9,10,11,12], wherein deaths cluster by location but not time, and are known as ‘locations where people frequently take their lives’, often occurring at well-known public or historical sites; however, this pattern is not as extensively researched compared with mass and point clusters. A phenomenon known as echo clusters, wherein one or more successive suicide cluster occurs at a distinct point away from the initial cluster, has been statistically verified in indigenous populations in rural Australia, but there is a dearth of evidence of this phenomenon elsewhere [14,15]. 

In recent years, an increasing number of studies have addressed the identification and detection of suicide clusters at both a national and local level [16,17,18]. The detection of clusters enhances the knowledge on the aetiology of emerging suicide clustering by establishing links between confirmed or suspected suicide cases and identifying socioecological factors associated with the increased risk of clusters within the affected area or population [19]. Policy makers and public health officials also benefit from early detection of suicide clusters by means of implementing targeted and timely interventions. Significant advances in spatial cluster detection have emerged in recent decades with the development of computer mapping and its integration with robust statistical models [19]. Previous studies that investigated the presence of suicide clusters have applied different techniques that follow frequentist and Bayesian probability models, and they have incorporated spatial scanning software [16,17,18,19,20]. Nonetheless, a standardized and systematic approach to the statistical ascertainment of suicide and self-harm clusters is still lacking in contemporary research. 

To date, no systematic review of the quantitative methods that effectively detect suicide and self-harm clusters has been conducted. The main aim of this systematic review is to synthesize the existing evidence based on statistical techniques used in successfully detecting suicide and self-harm clusters. In this regard, this review seeks to determine an accurate and precise approach to quantitatively verify suicide and self-harm clusters within a population, and to ensure that clusters of suicide and self-harm are detected in a timely manner, hence mitigating further cases. 

## 2. Materials and Methods

In accordance with the PRISMA guidelines [21], a comprehensive search strategy was established, including MeSH terms where relevant (see supplementary material for completed PRISMA checklist). The review was registered with The International Prospective Register of Systematic Reviews (PROSPERO, registration number CRD42018100354) to avoid duplication. The search strategy was applied to four bibliography databases: Medline, Embase, Web of Science, and Scopus from their inception to August 2018, to identify as much relevant literature as possible. The lead author conducted an updated search, applying the same search strategy in December 2020. The search terms included ((suicide (MeSH) OR suicid*) OR (self-injurious behaviour (MeSH) OR (self-injur* OR self-poison* OR self-mutilat* OR self-harm*)] AND [(cluster* OR imitat* OR contagion OR copycat OR werther effect)] OR (spatiotemporal analysis OR time-space analysis OR geospatial analysis OR statistical analysis*)). Inclusion criteria included studies that (a) have been published in a scholarly journal, (b) have applied a statistical method to detect suicide or self-harm clusters in a population, and (c) have the full-text available in English. Exclusion criteria eliminated (a) narrative reports of suicide or self-harm clustering that were not statistically verified, (b) grey literature including media reports relating to potential suicide or self-harm clusters, and (c) non-English language articles.

The title and abstracts of all references generated by the search were screened for relevance by three authors (RB, GC, LST) to avoid content bias. For those articles of which full texts were not available, the full text was requested from the lead author. Additional hand searches of reference lists of relevant systematic reviews were also conducted to identify other eligible studies. Only published scholarly articles were included to obtain the most robust methodological approach possible. Data extraction in table format was used to summarize study results. A meta-analysis was not considered due to the heterogeneity of statistical methodology applied in the included studies; hence, the data was narratively synthesized as a result. Subgroup analysis was conducted on four study groups based on commonalities in cluster type identified during preliminary analysis. For the purpose of the current research and to avoid misinterpretation, suicide clusters will henceforth refer to clusters of death by suicide, whereas self-harm clusters will describe the clustering of self-harm events including attempted suicide. 

## 3. Results

**Figure 1:**
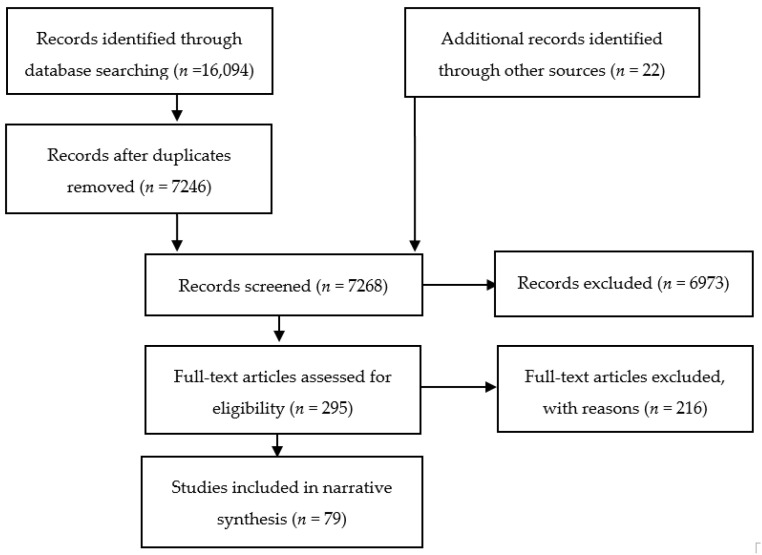
PRISMA flow diagram.

The electronic searches identified 7246 publications, excluding duplicates. Based on the screening of titles and abstracts generated by the database searches, 295 potentially relevant publications were identified. Of those publications selected for full text screening, 216 did not meet the eligibility criteria, resulting in 79 relevant articles applicable for review (Figure 1; full details of all relevant articles included as supplementary material). The relevant studies were sub-divided, based on their primary focus, into point suicide clusters (*n* = 51), point self-harm clusters (*n* = 8), mass suicide clusters (*n* = 19), and echo suicide clusters (*n* = 1).

The literature in this area predominately originates from the Oceania continent, Europe, and the Americas. Considerably less research on the topic has been published in Asia and in the African region to date. In terms of the level of geographic samples analyzed, approximately half of all studies were based on a national sample (*n* = 39), almost a third involved a regional sample (*n* = 25), and the remaining studies focused on state, city and investigations into locations associated with frequently occurring suicides or self-harm acts. The statistical analysis of point suicide and self-harm clusters commenced in 1975, with over two thirds of studies published in the last 5 years (*n* = 35). Although mass suicide cluster statistical detection was first documented within the literature in 1986, almost two thirds of studies have been conducted within the last 5 years (*n* = 12).

The majority of identified publications (*n* = 51) primarily focused on the statistical analysis of point suicide clusters (Table 1). Those studies with alternative primary objectives predominantly evaluated clusters in the context of their association with demographics, socio-economic factors, cultural variables, and risk factors associated with suicide clusters [7,22,23,24]. A Poisson model incorporating the Monte Carlo simulation is the most widely applied statistical model, applied in half of all point suicide cluster detection studies (*n* = 28) [16,17,22,24,25,26,27,28,29,30,31,32,33,34,35,36,37,38,39,40,41,42,43,44,45,46,47,48,49]. This test determines the significance of likely clusters, most commonly at a 5% spatial parameter and three-month temporal parameter, as well as detecting the relative risk of cluster. Geospatial analysis was conducted in three quarters of point suicide cluster detection studies (75%, *n* = 38), by means of spatial scanning using SaTSCan and FleXScan software, as well as hotspot analysis, spherical trigonometry, and Nearest Neighbor Analysis Exploratory Spatial Data Analysis methods [16,17,18,22,23,25,26,27,28,29,30,31,32,36,37,38,40,41,42,43,44,45,46,47,50,51,52,53,54,55]. ArcGIS was the most predominantly employed data visualization tool, whereas a small number of studies utilized alternate tools such as QGis, GeoDa and Teraview. A minority of studies applied alternative statistical analyses, including a regression model (*n* = 10) [22,26,29,38,43,44,50,56,57,58,59], the Knox procedure (*n* = 1) [7], descriptive network analysis (*n* = 1) [47], Bayesian hierarchical modelling (*n* = 2) [24,25,48,49,59,60], the Kernel Density estimator (*n* = 2) [51,58], Nearest Neighbor Analysis (*n* = 1) [50], Ripley’s K function (*n* = 1) [52], Moran’s I (*n* = 4) [50,59,60,61], chi square (*n* = 5) [50,51,55,62,63], Fisher’s exact test (*n* = 1) [64], and the Anderson Darling test (*n* = 1) [65]. Specific numerical details of detected clusters were undocumented in the results of those studies that applied alternative statistical techniques without the application of geospatial analysis. Comparing different statistical methods, a discrete Poisson-based scan statistical approach will capture most parameters to identify point suicide clusters, since suicide mortality data tends to be in a Poisson distribution.

A small number of studies (*n* = 8) focused on the detection of self-harm clusters within populations (Table 2). Almost a third of self-harm cluster detection studies were based on national samples (*n* = 3), with the remainder focusing on cluster detection at regional (*n* = 2), county (*n* = 1), and city levels (*n* = 2). Most studies reported a significant detection of self-harm clusters (*n* = 7), with over half of the studies (*n* = 5) indicating the specific number of self-harm clusters detected within the population, ranging from one to twenty-five clusters. Scan statistics were applied in over half of all self-harm cluster detection studies [66,67,68,69], with an alternative temporal scanning method applied in one investigation [70]. Those studies that excluded geospatial techniques from the statistical analysis applied a regression model or chi-squared test [71,72,73]; however, detailed information relating to identified clusters was not explicated from such analyses. Based on a comparison of the statistical methods applied, a regression-based scan statistical model will capture most parameters to detect point self-harm clusters.

Within the identified studies, over one third (35%, *n* = 19) reported on mass suicide clusters (Table 3). Over two-thirds of mass suicide cluster research were based on national samples (68%, *n* = 13), relating to high-profile suicides reported within the media in their countries. The remaining studies investigated mass clustering with regional (*n* = 4), provincial (*n* = 1), and continental samples (*n* = 1). The primary aim of all the identified studies was the statistical verification of increased suicides within a population (i.e., the detection of mass clusters). The most employed statistical analyses include a time-series model such as the Seasonal Autoregressive Integrated Moving Average (SARIMA) model (*n* = 11) [74,75,76,77,78,79,80,81,82,83], a regression model (*n* = 8) [82,83,84,85,86,87,88,89], a Poisson model (*n* = 4) [84,87,88,89], and non-parametric tests (*n* = 3) [87,90,91]. When comparing statistical models to detect mass clusters, a time-series regression model will capture the parameters of mass clustering as accurately as possible, based on temporal data.

One study based on the statistical analysis of echo clusters, conducted in Australia, was identified within the literature [92]. The application of a Poisson scan statistic method to data based on the same geographical area but from two different periods, effectively detected several clusters in each period. Although there are no additional studies of this kind to compare this methodological approach against, the identified literature applies the same methodology as point suicide clusters with an additional time dimension.

## 4. Discussion

This systematic review provides unique insights into the scope of quantitative methods used to detect suicide and self-harm clusters. The findings of this review indicate that quantitative analysis of suicide and self-harm clusters continues to advance, in line with enhancements in statistical models of verification and spatial scanning methods. Developments in geographical cluster detection have coincided with a greater availability of spatial data [93]. Open-source Geographical Information System (GIS) software was applied in all but one identified study of spatial suicide clusters, offering strengths including cost-effectiveness, reproducibility, online support forums, and tutorials [94]. 

As corroborated by the results of the current review, the quality of geographical data captured in a GIS database is crucially important for geospatial analysis and depends on positional and attribute accuracy (e.g., latitude and longitude coordinates and health outcome), as well as completeness of data. An awareness of the specific criteria for what constitutes suicide and self-harm acts, the importance of data completeness, and the precision required in the measurement of geographical coordinates are all critical components of accurate data recording, and in turn, accurate cluster detection [95]. The vast majority of research involved retrospective ecological studies of suicide or self-harm clusters based on aggregated geographical, mortality, and census data. The implementation of active surveillance involving proactive contact with data providers to access, record, and complete, accurate and timely public health data, including geographical identifiers [95,96], is recommended to enhance the precision of cluster detection.

To date, probabilistic model-based spatial scan statistics are the most widely applied and reliable methods employed in the detection of point suicide and self-harm clusters. Despite a dearth of literature within the area, research investigating echo clusters of suicide has followed the same quantitative methodology as point cluster detection (i.e., a Poisson based spatial scan statistic), integrating an additional time dimension to account for analysis of at least two different time periods. The Poisson approach models how many times the event is likely to occur within a specific period, whereas the Monte Carlo simulation is used to evaluate the statistical significance of the likelihood ratio for each circle. Based on the significance test, the scan statistic can identify the most likely cluster, as well as secondary clusters, for which the likelihood ratios are less, but are still of importance [97]. 

SaTScan, which is a type of software using a cylindrical scan statistic involving a moving circular geographical-based scan window and a time-based height dimension of continuously varying radii, appears to be the most used scan approach within the reviewed literature [20]. This tool evaluates the statistical significance of point clusters with no prior assumptions of the data. Although this software has been extensively applied within epidemiological studies, a limitation of SaTScan is its inability to detect non-circular shaped clusters or hotspots, such as the shapes of roads or rivers [98]. 

To detect irregularly shaped clusters, alternative approaches have been proposed and applied within the reviewed research [98,99,100]. FleXScan [53], based on an adjustable spatial scan window, is effective in detecting clusters that assume arbitrary shapes [100,101,102,103]; however, the efficacy of this software is limited to the detection of small to moderate clusters of approximately 30 cases [53]. Echelon scanning using EcheScan, also identified within the literature, is used to detect non-circular shaped hotspots based on their spatial hierarchal structure, visually represented by a dendrogram that is scanned from top to bottom [97,102]. Similar to the traditional spatial scan statistic, echelon scanning is based on the Poisson model with Monte Carlo simulation; however, the scan window is smaller. EcheScan software, developed in R, is easily accessible and incorporates open-source mapping tools; however, limitations exist in some instances wherein the shape of the detected hotspot may be too complex, or too large, to be easily interpreted [103].

The results of this review suggest that analysis window parameters of scan statistic algorithms should be manipulated to determine the appropriate population and duration thresholds, calibrating the optimal parameter combination, since the precision of results can be affected by scale. Future research should seek to compare the performance of the scan statistic algorithms via a simulation study and examine the spatial congruence and sensitivity of the models. Based on the unique purposes of the scan statistics, the robustness and sensitivity of a Poisson-based spatial scan hybrid approach should also be explored by future research. 

Mass cluster detection fundamentally concerns itself with an increase in cases during a specific period, irrespective of spatial relevance. Quasi-experimental research designs, such as time-series forecasting based on a regression model, measure how many future observations are predictable based on past behavior [85,104]. In mass cluster detection, media coverage of a fictional or real high-profile suicide is correlated with an increase in cases of suicide during the aftermath of the suicide, by means of comparing frequencies of suicide in an experimental time frame during and after the death was reported, against the frequency of suicide in a control period. Such studies involve a crucial limitation that must be considered when interpreting findings; that is, the difficulty to accept observed increases in suicide and self-harm rates in terms of being a direct link to the high-profile case with absolute confidence.

### 4.1. Strengths and Limitations

This review sought to identify and synthesize literature relating to suicide and self-harm cluster detection, demonstrating inclusivity in systematically reviewing all published studies to date, and addressing all types of suicide and self-harm clusters. The primary focus of the review was to examine the most robust global evidence using statistical methods to detect suicide and self-harm clusters within a population as accurately as possible; therefore, non-peer reviewed reports have been excluded from the synthesis, which may limit the results. Due to study heterogeneity arising from methodological diversity, a full quality appraisal was not carried out, hence, possible biases must be considered in the context of limitations. Excluding non-English studies has not limited the review since most research in this area has been conducted in English speaking countries.

### 4.2. Implications for Suicide Prevention and Considerations for Future Research

The findings of this review have implications for suicide prevention. More specifically, this review has synthesized all empirical studies of suicide and self-harm clusters in a population, arriving at the most comprehensive standardized approach to suicide and self-harm cluster detection currently available, in the absence of a gold-standard method. Innovatively, the conclusive approach of geospatial probabilistic modelling for point suicide cluster detection has been incorporated in the development and evaluation of a community response to a suicide cluster, demonstrating the utility of this technique for suicide prevention purposes [44]. The comprehensive study identified in the review applied spatiotemporal analysis to suicide mortality data and socioeconomic aggregated data by way of identifying suicide clusters and spatial variations of risk-factors in Hong Kong, for the purpose of informing the development of the targeted program, and evaluating its efficacy post-program, using changes in suicide incidence and cluster patterns as the outcome. The findings of the study emphasize the value of a temporal and spatial monitoring surveillance system based on the methodology described here in prioritizing suicide prevention measures. The outcome of the novel study further suggests a use for such techniques in the monitoring and evaluation of population-level interventions to be implemented as components in national suicide prevention strategies.

Official suicide mortality records can take up to two years post-death to be released, due to delays resulting from prolonged medico-legal cause of death investigations, and late registered deaths [105]. The application of cluster detection methods identified in this review to provisional, real-time, suspected suicide data, would support the detection of emerging clusters, providing an advanced opportunity to effectively intervene and mitigate further contagion [106]. Early identification of emerging suspected clusters would also facilitate the acceleration of an evidence-based crisis response in vulnerable communities, wherein screening and referral of susceptible individuals to appropriate clinical and support services could occur in a timelier manner. Future research should consider the investigation of self-harm clusters and suicide clusters within a population, to determine whether clusters of self-harm precede clusters of suicide, thereby offering the opportunity for targeted clinical intervention in populations wherein emerging self-harm clusters are detected as a prevention strategy for possible subsequent suicide clustering. 

Real-time active surveillance of suicide and self-harm would facilitate prospective studies of suicide and self-harm clusters using prospective geospatial probabilistic modelling [107]. The findings of such prospective studies would subsequently inform suicide prevention strategies, action plans, policy planning, and service provision in a timely manner. Although unexplored in studies, including those in this review, temporal analysis of suicide data using a calendar approach based on date of death may detect temporal clusters relating to significant dates, such as the anniversary of the death of a loved one or high-profile individual, and seasonal trends when peaks are commonly observed. The detection of this phenomenon should be incorporated as a key objective of a real-time suicide surveillance system by way of indicating high-risk dates and periods that could require deployment of additional resources to respond to possible increases in imitative behavior. 

## 5. Conclusions

The synthesized results of this systematic review demonstrate advances made in epidemiological cluster detection, which is relevant to suicide and self-harm data, within the forty-five-year period since statistical investigations into clusters of suicide and self-harm were first published. Most notably, the evolvement of open-source GIS software, has effectively contributed to point cluster detection by means of geospatial probabilistic modelling. Mass suicide cluster detection traditionally employs a time-series regression analysis to verify temporal clustering within a population; however, the use of retrospective aggregated data in these studies compromises the accuracy and efficiency of cluster detection investigations.

## Figures and Tables

**Table 1:** Point suicide clusters.

Number of Studies	51												
Level of data used in the study	Location			City			State		Regional			National	
	2			2			6		18			23	
Type of analysis (Studies that performed multiple statistical analyses are counted twice.)	Nearest Neighbour Statistic	Kernel density estimator	Spherical Trigonometry	Descriptive network analysis	Knox procedure	Ripleys k function	Chi-square	Fishers Exact Test	Morans I	Bayesian hierarchical model	Anderson Darling	Regression model	Poisson model
	1	2	1	1	1	1	5	1	4	6	1	10	28
Geospatial analysis conducted	No					Yes							
	13					38							
Clusters detected	No					Yes							
	3					48							
Addressed analysed	Not specified		Location of death					Residence					
	4		13					34					
SaTScan spatial applied	Not specified		No					Yes					
	7		24					20					
Number of clusters reported	Not specified			20+			1–20				No clusters detected		
	7			3			28				3		

**Table 2:** Point self-harm clusters.

Number of Studies	8							
Level Of Data Used in the Study	National	National	Regional	City	Regional	City	National	County
Location of studies	Sweden	New Zealand	New South Wales, Australia	Edinburgh, Scotland	Kwai Tsing, Hong Kong	Hamadan, Iran	Denmark	Meru, Kenya
Aggregated data used	No	No	Yes	No	Yes	No	Yes	No
Type of statistical analysis conducted	Logistical regression	SaTScan and Knox Procedure	SaTScan, Hotspot analysis (Getis-Ord Gi*) and ArcGIS for mapping	A scan interval test proposed by Naus, 1966	Chi square and SaTScan	Logistic regression and Chi-square, SaTScan and Monte Carlo simulation	Multi-level regressions and log likelihood ratio tests	Multiple logistic regression
Geospatial analysis conducted	No	Yes	Yes	No	Yes	Yes	No	No
Clusters detected	Yes	Yes	Yes	Yes	Yes	Yes	No	Yes
Number of clusters reported	Not specified	Not specified	Twenty-five spatial cluster regions identified	1 cluster	Four spatial clusters, one spatiotemporal cluster	2 clusters	*N*/A	Not specified

**Table 3:** Mass clusters.

Number of Studies	19			
Level of data used in the study	Continental	National	Regional	Provincial
	1	13	4	1
Type of analysis conducted (Studies that performed multiple statistical analyses are counted twice.)	Poisson model	Regression analysis	Non-parametric tests, i.e., Mann–Whitney U test, Kolmogorov-Smirnov test	Time-series models, e.g., SARIMA
	4	8	3	11
Geospatial analysis conducted	Yes		No	
	0		19	
Mass cluster(s) detected	Yes		No	
	17		2	

## Data Availability

Not applicable.

## Supplementary Materials

The following supporting information can be downloaded at: https://www.mdpi.com/article/10.3390/ijerph19095313/s1, Table S1: PRISMA 2009 Checklist; Table S2: Point Suicide Clusters; Table S3: Point Self-Harm Clusters; Table S4: Mass Clusters; Table S5: Echo Clusters.
