# Supplementary material for: Quantitative Methods to Detect Suicide and Self-Harm Clusters: A Systematic Review
Source: Int J Environ Res Public Health. 2022 Apr 27;19(9):5313. doi: 10.3390/ijerph19095313 (PMC9099648; doi:10.3390/ijerph19095313)
Supplement: Supplementary file 1 [file ijerph-19-05313-s001.zip › Supplementary file S1. articles for inclusion.pdf]

Additional file 1. Table of included articles

Table S2. Point Suicide clusters

Authors	Year	Title	Level of suicide data<br><br>Source of data<br><br>Period of analysis	Aggregated data (Y/N)	Primary or secondary aim of the study	Type of analysis	Geospatial analysis (Y/N)	Level of geographical data	Clusters detected (Y/N)	Number of clusters detected
**Milner, Too & Spittal**	2018	Cluster Suicides Among Unemployed Persons in Australia Over the Period 2001-2013	National- Australia retrieved from the National Coroners Information System.<br><br>2001-2013<br><br>13-year period	No	Yes	**Poisson discrete** scan statistic<br><br>**Monte Carlo** Stimulation employed, p<0.05.<br><br>Clusters were scanned using the Binomial model, with restricted likelihood ratio and max cluster number 15 (default).	Yes<br><br>Recognising limitation of SaTScan, **FlexScan** was applied to identify irregular shaped clusters.	Residency area	Yes	13-year period: Spatial-temporal clusters: 10 clusters detected, accounting for 1.7% of all unemployed suicides. Spatial clusters: 12 spatial clusters detected, accounting for 13.4% of all unemployed suicides. Temporal clusters: 7 clusters detected, accounting for, 4.4% of all unemployed suicides.
**Gibbons, Clark and Fawcett**	1990	A statistical method for evaluating suicide clusters and implementing cluster surveillance	Regional – Cook County, Illinois. 1977-1986<br><br>10-year period	No	Primary	**Poisson mixture model**	No	Not specified	No	N/A
**Bando, Moreira, Pereira & Barrozo**	2012	Spatial clusters of suicide in the municipality of Sao Paulo 1996-2005: an ecological study	Regional– Sao Paulo, Brazil.<br><br>Data were obtained from the Death Records Improvement Program, the official health statistics source for the Municipality.<br><br>1996-2005<br><br>10-year period	Yes – mortality and population data.	Primary.<br><br>Secondary aim to verify possible associations between the suicide risk clusters and socio-economic and cultural variables.	**Poisson distribution** Statistical significance of a given cluster was ascertained by the **Monte Carlo** procedures.	Yes<br><br>**SaTScan**	Residency area	Yes	Two significant spatial clusters were identified. The first was of increased risk (RR=1.66), comprising 18 districts in the central region. The second cluster detected was of decreased risk (RR=0.88), including 14 districts in the southern region.

**Bando, Moreira, Pereira & Barrozo**	2012	Suicide rates and income in Sao Paulo and Brazil: a temporal and spatial epidemiologic analysis from 1996 to 2008	National –Brazil and Regional- Sao Paulo<br><br>Data obtained from the Brazilian and São Paulo State Death registry databases.<br><br>1996-2008<br><br>13-year period	Yes – ecological data	Secondary.<br><br>The primary objective of this study is the suicide rates and income in Sao Paulo and Brazil: a temporal and spatial epidemiologic al analysis.	*Temporal analysis*: **Joinpoint Regression** to identify and estimate points of inflection in suicide rate trends. *Spatial analysis*: The suicide RR of each spatial unit was caulated using a **Poisson model.** Statistical significance of a given cluster was ascertained using **Monte Carlo** procedures.	Yes<br><br>**SaTScan**	Not specified	Yes	Two significant clusters. One cluster of high rate suicide and one of low rate suicide. The former cluster was located in the South region, encompassing 73 micro-regions; the latter cluster includes 98 micro-regions and comprises the east border.
**Reser**	1989	Australian Aboriginal Suicide Deaths in Custody- Cultural Context and Cluster Evidence	National – Australia. (based on Aboriginal population) Data obtained from the Royal Commission.<br><br>1980-1988.<br><br>9-year period	Yes	Primary	A **chi square** test was applied based on geographic and demographic information.	Yes<br><br>no scan or GIS software applied.	Location of death - prison	Yes<br><br>'A chi-square test strongly supports a non-random dispersion of the deaths over time.'	One – findings suggest strong support for a cluster interpretation of at least eight deaths in Queensland.
**Ngui, Apparicio, Moltchanova, Vasiliadis**	2014	Spatial analysis of suicide mortality in Quebec: Spatial clustering and area factor correlates	Regional – Quebec province in Canada.<br><br>Data obtained from Quebec's death registry.<br><br>2004-2007.<br><br>4-year period	Yes – the ecological data.	Primary	**The Bayesian hierarchical modelling** using GeoBugs1.4.3. and **logistic regression** with SAS 9.3. Monte Carlo test of likelihood.	Yes<br><br>**SaTScan**	Residency area	Yes	Clusters of suicide were located in 194 FSAs (Forward Sortation Area, the territory covered by the first 3 letters of a postcode). Analyses by gender showed a higher number of clusters of suicide in men than women.
**MacKenzie, Lester, Mansen & Yeh**	2016	Do Suicides From the Golden Gate Bridge Cluster?	Location based – Golden Gate Bridge, San Francisco. Data for Marin County accessed from the medical examiner's office. 1999-2009 10-year period 224 cases from GGB compared against 343 cases of other methods of suicide	No	Primary	The Anderson-Darling test was run against a null hypothesis of a negative exponential distribution	No	Location of death	No.	N/A

			in the county during the same time period.							
**Kirch & Lester**	1986	Suicide from the Golden Gate Bridge: Do they cluster over time?	Location based – San Francisco suspension bridge.<br><br>1976-1977<br><br>2-year period<br><br>47 jumpers were recorded.	No	Primary	The **Poisson distribution** model.	No	Location of death	No. No discernible clustering effect was apparent.	N/A
**Larkin & Beautrais & Xu**	2010	Geospatial mapping of suicide clusters in Canterbury	Regional – Canterbury, New Zealand<br><br>Data obtained from the New Zealand Ministry of Health<br><br>1991-2008<br><br>18-year period	No	Primary	Latitude and longitude coordinates for the residential street address for each decedent were geocoded using **Google maps**.	Yes<br><br>**SaTScan**<br><br>Google Maps used to geocode	Residency area	Yes	Nine suicide clusters involving 43 individuals (4.7% of all suicides).
**Niemi**	1975	The time space distances of suicides committed in the lock up in Finland in 1963-1967	National – Finland. Data based on deaths in jail.<br><br>1963-1967.<br><br>4-year period	No	Primary	Epidemiological methods were applied to determine the presence of clustering within prison in Finland.	No	Location of death	Yes	One - Clustering in terms of time (24-48 hrs after a suicide) was found to be statistically significant.<br><br>Clustering in terms of space was not found.<br><br>27 of 28 suicides were by hanging.
**Pérez-Costillas, Blasco-Fontecilla, Benítez, Comino, Antón, Ramos-Medina, Lopez, Palomo, Madrigal, Alcalde, Perea-Millá, Artieda-Urrutia, De León-Martínez, De Diego Otero**	2015	Clusters de casos de suicidio espacio-temporal en la comunidad de Antequera (España)	Regional – Malaga, Spain.<br><br>The Institute of Legal Medicine, Málaga.<br><br>2004-2008<br><br>5-year period	No	Primary	A spatiotemporal permutation scan statistic using **SaTScan** and the **Ripley's K function** were used to explore spatiotemporal clustering. **Pearson's chi-squared** was used to determine whether there were differences between suicides inside and outside the spatiotemporal clusters.	Yes<br><br>SaTScan	Residency area	Yes	7 spatio-temporal statistically significant clusters were detected.

**Austin, Amy E. van den Heuvel, Corinna Byard, Roger W**	2011	Cluster hanging suicides in the young in South Australia	Regional – South Australia.<br><br>Data obtained from Forensic Science South Australia<br><br>Two 5-year periods: 1995–1999 and 2005–2009	No	Primary	Statistical analyses were performed utilizing **Fisher's exact tes**t to compare the distribution of hanging suicides	No	Location of death	Yes	Data revealed a significant increase in hanging suicides in the young in South Australia in recent years. 14 deaths by hangings took place in recent times in specific area.
**Carcach, Carlos**	2017	A spatio-temporal analysis of suicide in El Salvador	National – El Salvador.<br><br>Data obtained from Institute of Legal Medicine for each of the 262 Salvadorian municipalities.<br><br>2002-2012<br><br>10-year period	No	Secondary – the main objective of this study was to examine completed suicide and homicide using the stream analogy to lethal violence within a spatio-temporal framework.	A **Bayesian model** was applied to examine the spatio-temporal evolution of suicide. WinBUGS were used to employ both models **Monte Carlo** test of likelihood employed at 5%.	No	Not specified	Yes	The results suggest the presence of time-persistent clusters of suicide risk among north-eastern and centre -southern municipalities.<br><br>Breakdown of numbers not available within text.
**Ceccato & Uittenboga ard.**	2016	Suicides in commuting railway systems: The case of Stockholm county, Sweden	Regional – Stockholm, Sweden.<br><br>2006-2013<br>120 suicides<br><br>7-year period	No	Primary	**Nearest Neighbour Hierarchial Cluster** (NNHC) and **Getis-Ord** statistics (Hot Spot analysis). **Regression models** – Chi square Pearsons 0.5 ratio.	Yes<br><br>GeoDa	Location of death (85% coordinates info, 15% free text).	Yes	5 significant clusters were detected using NNHC.<br><br>No temporal or seasonal clusters were detected.
**Cheung, Spittal, Williamson, Tung, Pirkis.**	2013	Application of scan statistics to detect suicide clusters in Australia	National – Australia. Data obtained from the National Coroners Information System (NCIS).<br><br>2004-2008<br><br>4-year period	Yes	Primary	**A Poisson Discrete model** of scan statistics was applied. Statistical significance of cluster evaluated by **Monte Carlo** test.	Yes<br><br>**SaTScan**<br><br>**ArcGIS** used to form a digital boundary map.	Residency area	Yes	15 clusters were detected overall. 4 (26.7%) were detected by both national and state cluster detections, 8 (53.3%) were only detected by the state cluster detection, and 3 (20%) were only detected by the national cluster detection.
**Cox & Skegg.**	1993	Contagious suicide in prison and prison cells	National – New Zealand.<br><br>Data obtained from the Department of Justice, NZ.	No	Primary	The event of suicide was treated as a **Poisson process** with all suicides in a time period used to calculate the	No	Location of death	Yes	Four suicide clusters occurred between 1985 and 1988 in prisons. Three clusters of suicide in police cells occurred between 1980 and 199

			Suicide in Prisons: 1971-1988<br><br>18-year period<br><br>Police cells: 1980- 1991.<br><br>11-year period			mean. If the probability of the number of suicides observed within a short time period was less than 5% a cluster was considered likely.				
**Dantas, Azevedo, Nunes, Amador, Marques, Barbosa.**	2018	Analysis of suicide mortality in Brazil: spatial distribution and socioeconomic context	National – Brazil<br><br>Mortality Information System of the Brazilian Unified Health System<br><br>2010-2014<br><br>4-year period	Yes	Primary	The spatial distribution, intensity and significance of the clusters were analysed with the global **Moran index**, MoranMap and local indicators of spatial association seeking to identify patterns through geostatistical analysis. **Bivariate LISA analysis** was performed.	Yes	Location of death –municipality	Yes	High mortality cluster formations were detected in the South. No breakdown of numbers provided.
**Exeter & Boyle**	2007	Does young adult suicide cluster geographically in Scotland?	National – Scotland. Data provided by the General Register Office for Scotland for<br><br>three independent 3 year periods:<br><br>1980- 1982, 1990 – 1992, 1999 - 2001	Yes – deaths were aggregated into 10058 small areas of Scotland.	Primary	The spatial scan statistics was applied. A **Monte Carlo** simulation was used to test the significance of the likely clusters	Yes	Residency area	Yes	A significant geographical cluster of suicide among young adults was identified in east Glasgow in all three time periods (involving 92, 159, and 245 cases)
**Fontanella, Saman, Campo, Hiance-Steelesmith, Bridge, Sweeney, Root.**	2018	Mapping suicide mortality in Ohio: A spatial epidemiological analysis of suicide clusters and area level correlates	Regional – Ohio, USA.<br><br>Data retrieved from Ohio Department of Health.<br><br>2004- 2013.<br><br>10-year period	Yes<br><br>Ecological data was aggregated and geocoded to census tract level for analysis of area level factors.	Primary	Two separate spatial analyses – First, a spatial discrete **Poisson probability model.** Secondly, a space-time discrete **Poisson model** was employed to search for high rates using a 1-month time aggregation period.	Yes<br><br>Spatial displays made in **ArcGIS** 10.4.<br><br>All analyses were implemented in **SaTScan** software.	Residency area	Yes	Nine statistically significant high-risk spatial clusters and two space-time clusters were identified.
**Gould, Wallenstein, Kleinman,**	1990	Suicide clusters: an examination of age-specific effects	National – United States of America.	No	Secondary – main aim of study is to	**The Knox procedure** was	No	Residency area	Yes	Suicide clusters appear to occur primarily among teenagers and

**O'Carroll, Mercy.**			Data obtained from the National Centre for Health Statistics<br><br>1978 -1984<br><br>7-year period		examine the age-specific effects of suicide cluster	used to assess the degree of time-space clustering. The Knox procedure considers all possible pairs of cases and the time and space distances between them.				young adults, although clusters do occur occasionally at other ages. Breakdown in number of clusters not available.
**Johnson, Woodside, Johnson, Pollack.**	2017	Spatial Patterns and Neighbourhood Characteristics of Overall Suicide Clusters in Florida from 2001 to 2010.	State – Florida, USA.<br><br>Data obtained from Department of Health Bureau of Vital Statistics.<br><br>2001- 2010<br><br>10-year period	Yes – all suicides aggregated for each Census tract (CT) in the state of Florida.	Primary	The scan statistic method was used to detect suicide clusters. **Regression analysis** was used to investigate the association of neighbourhood characteristics with identified clusters. **Monte Carlo** test for likelihood ratios applied.	Yes<br><br>Identified clusters were mapped using GIS software.	Not specified	Yes	Twenty-four high-risk and 25 low-risk clusters were identified. The risk of suicide was up to 3.4 times higher in high-risk clusters than in areas outside of clusters.
**Jones, Gunnell, Platt, Scourfield, Lloyd, Huxley, John, Kamran, Wells, Dennis**	2013	Identifying probable suicide clusters in wales using national mortality data	National – Wales.<br><br>Data obtained from the Office for National Statistics<br><br>2000- 2009<br><br>10-year window	No	Primary	**Space Time Permutation Scan Statistics** (STPSS) was applied – it uses a Poisson based likelihood to compare the expected number of cases and actual number of cases inside and outside the window. **Monte Carlo** test for likelihood ratios applied.	Yes<br><br>spatial analysis was performed using Scan Statistics with **SaTScan**.	Residency area	Yes (possible)	One small possible cluster in young people between December 2007 and February 2008.
**Joo**	2017	Spatiotemporal study of elderly suicide in Korea by age cohort	National –South Korea.<br><br>Data obtained from the National Statistical Office of Korea.<br><br>2001-2005.<br><br>5-year period	Yes- to municipal level. It includes gender, marital status, education, cause of death, investing person, date of death and location of death (home,	Primary	The **Gi* score**, which is a spatially weighted indicator of area attributes, was used to identify hot spots and the spatiotemporal pattern of elderly suicide in the nation during the last 10 years. The Gi* index is a	Yes	Location of death	Yes	No explicit clusters of suicide detailed within the results of the study. Clustering as a whole is outlined within the populations 65-74 years and 75+. The suicide rates of people 75 years and older are approximately twice as high as those aged 65-74 years. Those

				hospital or assisted living centre)		spatially weighted indicator, displaying a high or low cluster of area attributes. The spatial **Markov matrix** and **spatial dynamic panel** data model were employed to identify and estimate the diffusion effect.				rates of age 65-74 years cluster under the suicide rate, 100,
**Qi, Hu, Page, Tong**	2012	Spatial clusters of suicide in Australia	National – Australia.<br><br>Data obtained from the Australian Bureau of Statistics<br><br>1999 to 2003.<br><br>5-year period	No	Primary	A **Poisson regression model** was performed to compute the mean relative risk (RR) of each cluster and likelihood ratio to identify the two types of clusters as circular windows.	Yes<br><br>GIS and mapping approaches were applied by means of **SaTScan**	Residency area	Yes	13 clusters detected, 1 primary male, 1primary female and 11 secondary male clusters.
**Qi, Tong & Hu**	2010	Spatial distribution of suicide in Queensland, Australia	Regional – Queensland, Australia.<br>Data obtained from Australian Bureau of Statistics<br><br>1999-2003<br><br>5-year period	Yes	Secondary	The suicide relative risk (RR) of each Local Governmental Area was calculated using a **Poisson model**, and the mean RR of each cluster was computed with the **SaTScan**	Yes	Residency area	Yes	Clusters were separated into most likely and secondary likely. One most likely cluster was detected and 6 secondary likely clusters were detected.
**Robinson J, San Too L, Pirkis J, Spittal MJ.**	2016	Spatial suicide clusters in Australia between 2010 and 2012: a comparison of cluster and non-cluster among young people and adults.	National – Australia.<br><br>Data obtained from the National Coronial Information System<br><br>2010 – 2012<br><br>3-year period	No	Secondary – main objective to compare suicide clusters and non-clusters among young people and adults.	Poisson discrete model employed, as this model is adjusted for the uneven geographical population density. The likelihood of each cluster was assessed using **Monte Carlo** stimulation.	Yes<br><br>Scan statistics were applied using SaTScan v 9.4.1. The geographical coordinates were calculated with **ArcGIS**.	Residency area	Yes	12 clusters detected, 5 among young people and 7 among older people.
**Strauss, Klimek, Sonneck, Niederkrotenthaler**	2017	Suicides on the Austrian railway network: hotspot analysis and effect of proximity to	National- Australia. Data obtained from the main Austrian railroad operator ÖBB (N =1170). Data on the railroad	No	Secondary. The main objective of this study is to identify hotspots of	**Poisson model** was applied: We model the suicide locations as a spatial point pattern generated	Yes – not scan statistics.<br><br>The locations of the facilities were obtained	Location of death	Yes	15 hotspots detected. These hotspots represent 0.9% of the total track length (5916 km/3676 miles) that account for up

		psychiatric institutions	network was obtained from the publicly available Euroglobalmap from 2014.<br><br>1988-2009<br><br>12-year period		suicides on the Australia rail network and determine the proximity to psychiatric institutions.	by an IPP with a spatially varying log-linear intensity function of the spatial variables: population density, psychiatric bed density, single vs multi track, total suicide rate and socio-demographic factors.<br>The analysis was performed using Matlab with Statistics and Machine Learning Toolbox.	by looking up their street addresses using QGIS with overlay data from Google Maps, OpenStreetMap or Geoland Basemap.			to 17% of all railway suicides
**Tidemalm, Runeson, Waern, Frisell, Carlström, Lichtenstein, Långström**	2011	Familial clustering of suicide risk: a total population study of 11.4 million individuals	National – Sweden.<br><br>Data obtained from 3 linked database: the Cause of Death Register, the National Impatient Register and the Multi-Generational Register.<br><br>1952–2003.<br><br>52 year period	No	Primary – familial clustering detection was main aim.	A matched case-control design was applied. Odds ratios / relative risks were calculated with conditional **logistic regression** by comparing suicide rates in each proband-relative dyad type with population control-relative dyads matched 1: 5 by relative category, gender, birth year and time at risk.	No	Residency area	Yes	Familial clustering of suicide is primarily influenced by genetic and also shared environmental factors. the risk among full siblings<br>was higher than that for maternal half-siblings, despite similar environmental exposure. Further, monozygotic twins had a higher risk than dizygotic twins and cousins had higher suicide risk than controls.
**Tomita, Kubota & Ishioka**	2015	Spatial Clustering Properties in the Temporal Variation of Suicide Rates/Numbers among Japanese Citizens: A Comprehensive Comparison and Discussion	National - Data based on deaths identified as suicides extracted from Vital Statistical Data for Japan from 1973-2009. Data was split by genders and broken down into 6 time periods, 4 of which were focused on: 1999-1992, 1993-1997, 1998-2002, 2003-2007.	Yes. Data were edited, updated and segregated by municipality and secondary medical zone.	Primary	Echelon scans were performed to identify regions with the highest-likelihood ratio of suicide as the most likely suicide clusters<br><br>A spatial scan statistical model based on the **Poisson distribution** was employed.	Yes – not SaTScan.	Location of death	Yes	Clusters were identified within all time periods and in all 6 regions. The results revealed that the number of suicides among men grew significantly as time progressed through the study. For men, regions near major<br>metropolitan areas were the major clusters for suicides, esp. in 1988–1992, 1993–1997<br>and 1998–2002, which saw very rapid

										increases in suicides in men. For women, regions near major metropolitan areas were detected as clusters in 1993–1997, 1998–2002 and 2003–2007.
**Too, Pirkis, Milner, Bugeja, Spittal.**	2017	Railway suicide clusters: how common are they and what predicts them?	Regional- Victoria, Australia.<br><br>Data obtained from the National Coronial Information System.<br><br>2001-2012<br><br>12-year period	Yes - the number of railway suicides by month of the death and residential postcode.	Primary	The **Poisson discrete scan statistic** was performed to identify railway suicides that occurred close together in space &/or time. A case–control design was used to compare clustered railway suicides with non-clustered railway suicides on a range of individual and neighbourhood factors.	Yes<br><br>ArcGIS was used to compute the geographical coordinates of the postcode centroids.<br><br>SaTScan employed	Location of death	Yes	Four spatial clusters were detected, accounting for 35% of all railway suicides.
**Too, Pirkis, Milner, Spittal.**	2017	Clusters of suicides and suicide attempts: detection, proximity and correlates	Regional – Western Australia.<br><br>Data obtained from Data Linkage Western Australia (WA), Department of Health, WA.<br><br>2000-2011<br><br>12-year period	Yes - number of suicide attempts and suicides by month of the event and residential statistical level area.	Primary	**Poisson discrete** scan statistics used to detect clusters. **Logistic regression** was then used to compare clustered attempts to non-clustered attempts to identify risk factors for an attempt being in a cluster.	Yes<br><br>**SaTScan** employed	Residency area	Yes	350 suicide attempts occurred within 7 clusters and 12 suicides occurred within 2 clusters. The suicide clusters were located within a larger but later suicide attempt cluster.
**Alarcao, Agnolo, Vissoci, Carvalho,Staton, de Andrade, Fontes, Pelloso, Nievola & Carvalho**	2019	Suicide mortality among youth in southern Brazil: a spatiotemporal evaluation of socioeconomic vulnerability	State- Parana, Brazil<br><br>Data obtained from the DATASUS Mortality Information System and the Brazilian Institute of Geography and Statistics, based on three age groups (15-19, 20-24, and 25-29 years).<br><br>1998-2002, 2008-2012	Yes	Primary aim – to conduct a spatial analysis of suicide deaths among young people aged 15-29 years.<br><br>Secondary aim - to evaluate their association with socioeconomi	The **Global Moran Index** was used to calculate global and local spatial autocorrelations. LISA clustering method applied to graphically depict spatial autocorrelation, LISA choropleth maps were plotted to identify significant spatial clusters.	Yes<br><br>Spatial data analysis conducted in **GeoDa** opensource software.	Residency area	Yes	Number and risk level of clusters not specified. Municipalities with high suicide mortality rates were identified within the three age groups of interest.

			two 5-year periods		c<br>determinants.				
**Kassem, Carter, Jonhson & Hahn**	2019	Spatial clustering of suicide and associated community characteristics, Idaho, 2010-2014	State- Idaho, USA<br><br>Data obtained from the Idaho Bureau of Vital Records and Health Statistics<br><br>2010-2014<br><br>5-year period	No	Primary	Geocoding conducted using AGGIE system.	Yes<br><br>SaTScan applied. ArcGIS used for geographical displays.	Residency area	Yes	10 clusters detected - a “most likely” cluster and 9 secondary clusters
**Nunez-Gonzalez, Lara-Vinueza, Gault & Delgado-Ron**	2018	Trends and Spatial Patterns of Suicide amongst Adolescents in Ecuador, 1997-2016	National -Ecuador<br><br>Data obtained from the National Institute of Statistics and Census. Adolescent deaths aged 10-19.<br><br>1997-2016<br><br>20-year period	No	Secondary. Primary was the study of trends and spatial patterns of suicide among adolescents in Ecuador	A **Poisson** discrete model was used. Primary and secondary clusters were detected through the **LLR** test. **Montecarlo** simulation used to calculate statistical significance of the clusters. Spatial distribution analysed using Global Morans I Index. Bayesian smoothing method and LISA by means of Local Morans I index used to detect hotspots, coldspots and spatial outliers.	Yes<br><br>**SaTScan** used to detect high-risk spatiotemporal clusters. GeoDa and QGIS used for mapping.	Residency area	Yes	One significant spatio-temporal cluster detected from 2011-2013 in 83 catons, encompassing 13 provinces.<br><br>Two significant spatial clusters detected from 2011-2016. The primary cluster included same catons as spatio-temporal cluster. The secondary cluster included 20 catons in 5 provinces.
**Sy, Shaman, Kandula, Pei, Gould, Keyes**	2019	Spatiotemporal clustering of suicides in the US from 1999 to 2016: a spatial epidemiological approach	National (10 states): Montana, Alaska, Wyoming, New Mexico, Nevada, Utah, Idaho, Colorado, Arizona, and Oklahoma, USA<br><br>Data obtained from the (WONDER) dataset.<br><br>1999-2016<br><br>18-year period	Yes- completed suicide data aggregated to every 6 months	Primary	A discrete Poisson model was employed to retrospectively detect spatiotemporal suicide clusters. The likelihood of each possible cluster was assessed using Monte Carlo simulations.	Yes<br><br>SaTScan was employed.	Residency area	Yes	15 clusters were detected in first 3 years
**Dos Santos, Lima**	2018	Spatial analysis and temporal trends of suicide mortality in	Regional – Sergipe, Brazil	Yes	Primary	The **Kernel density estimator**	Yes<br><br>Spatial	Residency area	Yes	Numbers of clusters not detailed.

**Guimarães, de Carvalho, Costa Viana, Lima Alves, Rodrigues Lima, Bezerra Santos, Aurélio de Oliveira Góes & Gomes Machado de Araújo**		Sergipe, Brazil, 2000-2015	data obtained from the Brazilian Mortality Information System<br><br>2000-2015<br><br>16-year period			was used to detect spatial clusters.<br><br>temporal trend analysis performed using a simple **linear regression model**	analyses were performed using **TerraView** software version.			Analysis revealed clusters in the north region and along the east coast of the state. Moreover, Kernel density analysis showed that the central-eastern region of the state is the area at the highest risk of suicide (areas with clusters in all temporalities analyzed)
**Lai, Law, Shum, Ip, & Yip**	2018	A community-based response to a suicide cluster: A Hong Kong experience.	Regional – Hong Kong, China.<br><br>Data obtained from the Coroner's Court<br><br>2010<br><br>1-year period	No	Primary	Exploratory Spatial Data Analysis (ESDA)techniques in GIS, with the **Poisson model** based on the analyses of all suicides in Hong Kong.	Yes	Residency area	Yes	One cluster detected<br><br>6 cases<br><br>May – August 2010<br><br>All individuals resided in the same housing estate
**Lazzarinil, Gonçalvesl, BenitesIII, Ferreira da SilvaIII, Tsuha, Icksang Kol, Rohrbaugh, Andrews, Crodall**	2018	Suicide in Brazilian indigenous communities: clustering of cases in children and adolescents by household	Regional- Dourados, Brazil<br><br>Data obtained from The Brazilian National Mortality Database (SIM) and the SIASI suicide surveillance database.<br><br>2003-2013<br><br>11-year period	Yes	Primary	The proportion of suicides that occurred in a cluster was compared for different age strata, using **Pearson's chi-squared test** and expressed in odds ratios, to determine if household clustering of suicide specifically affected children and adolescents relative to other age groups.	No	Residency area	Yes	There was a total of eleven household clusters identified – nine with two cases each and two with three cases.
**Hsu, Chang, Lee & Yip**	2015	Geography of suicide in Hong Kong: Spatial patterning, and socioeconomic correlates and inequalities	Regional– Hong Kong<br><br>Data obtained from the Coroners Court<br><br>2005-2010<br>6 year period	Yes	Primary	**Bayesian hierarchical** models, based on **Poisson assumption** and estimated using the **Markov chain Monte Carlo** methods implemented in WinBUGS.<br>Moran's I statistics	Yes<br><br>ArcGIS	Residential area	Yes	Number of clusters not detailed

						were used to calculate				
**Sugg, Woolard, Lawrimore, Michael & Runkle**	2020	Spatial clustering of suicides and neighbourhood determinants in North Carolina 2000 to 2017	State– North Carolina, USA<br><br>Data obtained from the North Carolina Department of Health and Human Services<br><br>2000-2017<br>17-year period	Yes	Primary	Descrete **Poisson Spatial Scan** Statistic with Monte Carlo simulations.<br>**Logistic regression** used to assess the association of clusters with community level determinants.	Yes<br><br>SaTSCan<br><br>ArcGIs for geocoded addresses	Residential address	Yes	Across all suicides, 12 significant spatial clusters were detected. Four significant within the adolescent and young adult population. Nine within the working-age demongraphic.<br>One within the elderly demographic.
**Too, Spittal**	2020	Suicide Clusters Among Top 10 High-Risk Occupations: A Study From 2001 to 2016 in Australia	National – Australia<br><br>Data obtained from the National Coronial Information System and census population data.<br><br>2001-2016<br>16 year period	Yes	Primary	**Discrete Poisson Scan Statistic with Monte Carlo simulations,** using prespecified temporal and spatial scan windows.<br>Cluster detection based on 10 high risk occupations, as indicated by suicide rates.<br>'Possible clusters' were determined by p≤0.10 and 'clusters' determined by *p*≤0.05. The spatial window was set at 1% of population at risk with 10km radius. The temporal window was set at 1-18months.	Yes<br><br>SaTScan<br><br>ArcGIS for geocoded addresses	Residential address	Yes	Four spatio-temporal clusters detected among high risk occuptations. The largest cluster occurred over a period of 13 months during 2004 and 2005. The remaining clusters occurred over a period of 5 months and involved three deaths each. Three spatial clusters among at risk occuptations, involving a minimum number of 2 and a maximum number of 7 cases in each cluster. Two temporal clusters detected among high risk occupations in 2007 and 2008, involving 9 and 122 cases respectively.
**Hill, Too, Spittal, & Robinson**	2020	Understanding the characteristics and mechanisms underlying suicide clusters in Australian youth: a comparison of cluster detection methods	National – Australia<br><br>Data obtained from the National Coronial Information System and census population data.<br><br>2006-2015<br>10 year period	Yes	Primary	**1.Discrete Poisson scan statistic with Monte Carlo simulations.** The time window was set to 1 week – 2 years. The spatial window was set to 10% of population at risk with 100km radius.'Possible clusters' were determined by p≤0.10 and 'clusters'	Yes<br><br>SaTScan	Statistical area level of residence (SA2), i.e. general purpose areas reprensenting communities that interact socially and ecomically. Avg. population 10,000	Yes	Variations in clusters detected by each approach applied.<br>Scan statistic method: 8 spatio-temporal clusters were detected in 6 states and territories, involving 69 individuals.<br>Coroners inquest method: Seven inquests into clusters were detected in 3 states and territories, involving 40 individuals.

						determined by p≤0.05<br>**2. Coroners inquests in suicide clusters**<br>**3. Descriptive network analysis** used to identify suicide clusters that comprised social links between three or more suicide descendants, as evidenced by narrative text from police and coroner's reports				Descriptive network analysis method: 11 clusters comprising 7 triads and 4 tetrads detected in 4 states and territories, involving 37 individuals. <50% of clusters were identified by more than 1 cluster method. Majority of overlap (4 of 8 clusters) was detected via coroners reports and network analysis. Spatial congruence between overlapping was low (<100% of SA2s)
**Yamaoka, Suzuki, Inoue, Isikawa & Tango**	2020	Spatial clustering of suicide mortality and associated suicide characteristics in Kanagawa prefecture, Japan, 2011-2017	Regional (Country subdivision)– Kanagawa, Japan<br><br>Data obtained from the National Vital Statistics of Japan<br><br>2011-2017<br>7 year period	Yes	Primary	**flexible scan statistic** (primary analysis)**, circular spatial scan statistic** and **Tango's test** using Disease Mapping System. Linear regression and conditional autoregressive models were used to adjust for covariates and for estimate regional effects.	Yes<br><br>SaTScan and FlexScan	Region of residency	Yes	Variations in clusters detected by each statistical approach applied.<br><br>Number of clusters not detailed
**Beringuel, Canuto, Cabral, Bonfim**	2020	Epidemiology and Spatiotemporal of Suicides in the State of Pernambuco	State- Pernambuco, Brazil<br><br>Data obtained from the Brazilian Mortality Information System<br><br>1999-2018<br>20 year period<br><br>Analyzed by decade	Yes	Primary	**Empirical Bayes method** used to smooth the crude mortality rates. **Moran's Index** used to analyze the global and local spatial autocorrelation. Moran Scatteplot used to identify critical or transition areas & Moran Map used to verify municipalities with statistically significant (5%) positive spatial correlation.	Yes<br><br>Terraview	Municipality of residence	Yes	Two critical clusters and one low level cluster were identified totalling 18 municipalties in the first decade and 29 municipalities in the second decade.
**Yoshioka, Hanley,**	2020	Geography of suicide in Japan:spatial	National – Japan	Yes	Primary	**Bayesian Hierarchical models, based on**	Yes<br><br>QGiS	Municipality of residence	Yes	Number of clusters not detailed

**Sato & Saijo**		patterning and rural-urban differences	Data obtained from the Ministry of Health, Labour and Welfare<br><br>2009-2017<br>9 year period			**Poisson regression models** were used to estimate smoothed suicide mortality rates for each municipality, investigating associations level of rurality/urbanity and suicide rates				
**Santos, Barbosa, Severo**	2020	Space-time analysis of mortality by suicide in the state of Rio Grande do Norte, Brazil, in the period from 2000 to 2015	State- Rio Grande de Norte, Brazil<br><br>Data obtained from the	Yes	Primary	**Moran's Index** used to analyze the global and local spatial autocorrelation**.** **The local Moran Index & Moran Map** used to verify municipalities with statistically significant (5%) positive spatial correlation.	Yes<br><br>Terraview	Municipality of residence	Yes	Number of clusters not detailed
**Guo, Chau, Chang, Woo, Wong, Yip**	2019	The geography of suicide in older adults in Hong Kong: An ecological study	Regional- Hong Kong, China<br><br>Data obtained from the Coroners Court<br><br>2006-2015<br>10 year period	No	Primary	**Bayesian hierarchical models** used to estimate the smoothed suicide mortality rate (SMR**) f**or each area based **on Poisson assumption. Models** were estimated using the **Markov chain Monte Carlo methods. Moran's I statistics** was calculated to test for spatial autocorrelations in the SMRs**. Local Moran's I** was applied to identify the clusters of the high SMRs.	Yes<br><br>ArcGis<br><br>GeoDa	District council/ Constituency area of residence	Yes	Number of clusters not detailed
**Vaz, Shaker& Cusimano**	2020	A geographical exploration of environmental and land use characteristics of suicide in the greater Toronto area	City- Toronto, Canada<br><br>Data obtained from the Emergency Medical Service	Yes	Primary	**Nearest Neighbor statistics** was used to calculate the degree of clustering**. T-test** was used to	Yes<br><br>ArcGis	Neighbourhood of residence	Yes	Number of clusters not detailed

			2002 and 2004<br><br>2 separate years			compare distance of incident to public locations of interest**. Chi-square** was used to test temporal variations. **The kernel density maps and point overlays** were used to visualize the distribution of incidents and features. Hotspot analysis conducted using **Global Moran's I-test** and **Getis-Ord Gi* statistic.**				
**Bando, Barrozo & Volpe**	2020	Geographical clusters and social risk factors for suicide in the city of Sao Paulo, 2006-2015: An ecological study	Regional– Sao Paulo, Brazil.<br><br>Data were obtained from the Death Records Improvement Program, the official health statistics source for the Municipality<br><br>2006-2015<br>10 year period	Yes	Primary	**Spatial scan test** was used to detect the geographical clusters. **Correlation and multiple regression techniques were used to estimate the association of socio-economic and cultural variables with suicide.**	Yes	District of residence	Yes	Three clusters were identified which are as follows: one of increased risk in downtown and two of decreased risk in the South and in the Southeas
**Hill, Spittal, Pirkis, Torok, Robinson**	2020	Risk factors associated with suicide clusters in Australian youth:Identifying who is at risk and the mechanisms associated with cluster membership	National- Australia<br><br>Data were obtained from the National Coronial Information System<br><br>2006-2015<br>10 year period	Yes	Secondary	Suicide clusters were identified from the free text of electronic police & coroners reports. The distance between index & exposed cases was estimated by calculating the shortest possible distance between SA2 centroids, i.e. **spherical trigonomitry**	Yes<br><br>Geosphere in R	Statistical area level (SA2), i.e. general purpose areas reprensenting communities that interact socially and ecomically. Avg. population 10,000	Yes	Links were identified between 117 young people (51 suicide clusters). 50% of young people died within 90 days of the index suicide.
**Lersch**	2020	Exploring the geography of suicide threats and suicide attempts: An application of Risk Terrain Modeling	City- Detriot, USA<br><br>Data obtained from Open Data Portal which contains all	Yes	Primary	Hotspot analysis using Getis-Ord Gi* statistic	Yes<br><br>ArcGis	Location	Yes	Number of clusters not detailed.

	911 calls relating to suicides in progress and suicide threats to the Detroit Police Department (DPD)<br><br>2016-2019<br>3 year period

Table S3. Point self-harm clusters

Authors	Year	Title	Level of self-harm data<br><br>Source of data<br><br>Period of analysis	Aggregated data (Y/N)	Primary or secondary aim of study	Type of analysis	Geospatial (Y/N)	Level of geographical data	Clusters detected (Y/N)	Number of clusters detected
**Mittendorfer-Rutz, Rasmussen, Wasserman.**	2008	Familial clustering of suicidal behaviour and psychopathology in young suicide attempters	National – Swedish registers linked to individuals born during time period<br><br>1968-1980<br><br>13-year period of individual data	No	Primary	**Logistical regression**	No	Residential address	Yes	Clustering amongst family members of self-harm patients.
**Smeeton & Wilkinson.**	1988	The identification of clustering in parasuicide	Regional– Edinburgh.<br><br>Data based on all patients admitted to Edinburgh Regional Poisoning Centre<br><br>1980-1981<br><br>2-year period	No	Primary	**A novel scan interval proposed by the author**	No	Residential address	Yes	Thirty-one patients were deemed eligible for analysis. Eight of the31 patients showed a clustered pattern of episodes.<br><br>It was proposed that at least 5 episodes during the 2 years were required to distinguish between chronic and clustered patterns, and that at least four episodes during the 3-month scan interval were required to define a cluster of repeated episodes of parasuicide.
**Torok, Konings, Batterham, Christensen.**	2017	Spatial clustering of fatal, and non-fatal, suicide in new South Wales, Australia: implications for evidence-based prevention	Regional – New South Wales, Aus.<br><br>Suicide mortality data from Australian Bureau of Statistics.<br><br>Self-harm data from NSW Admitted Patient Data Collection.<br><br>2005-2013<br><br>9-year period	Yes. To ensure sufficient numbers of suicide deaths within regions were available to enable a robust analysis; consequently, temporal cluster analysis was not possible.	Primary	**Hot Spot Analysis (Getis-Ord Gi*).**	Yes.<br><br>**ArcGis** and SaTScan	Residential location	Yes	Altogether, 25 primary and secondary likely LGA cluster candidate regions were identified, comprising mainly urbanised (i.e., higher density) coastal and metropolitan constellations.<br><br>Across NSW, 14.3% (N = 74) of SA2s identified as members of SaTScan suicide mortality clusters, 14.5% (N = 75) identified as suicide mortality Hot-Spots, 17.4% (N = 90) as members of SaTScan self-harm clusters, and, 23.8% (N = 123) as self-harm Hot-Spots. Comparison of the two scan statistics shows that Hot-Spot analysis identified more suicide clusters in rural or remote areas of NSW,62% of which identified as non-fatal intentional self-harm clusters.

**Gould, Petrie, Kleinman & Wallenstein**	1994	Clustering of attempted suicide: New Zealand National Data	National- New Zealand<br><br>Data obtained from the New Zealand Health Statistics Service.<br><br>1988-1990<br><br>3-year period	No	Primary	The **Scan statistic** and **Knox procedure** were employed for testing the significance of clusters in time and time-space, respectively.	Yes	Location of act of self-harm, based on hospital of treatment	Yes	Clustering of attempted suicides in time appears to be occurring only among teenagers and young adults in New Zealand, specifically those in the 15-19 and 20-24 years age groups. Clustering was detected in these age groups when 7, 14 and 30-day windows were applied in the analysis. Clear evidence for clustering of suicide attempts in time and space emerged for the 15-19, 20-24 and 25-34 year age groups. There was also some evidence of clustering among the 55-64 year olds.
**Pisinger, Hawton & Tolstrop**	2019	School and class level variation in self-harm, suicide ideation and suicide attempts in Danish high schools	National – Denmark<br><br>Data obtained from the National Youth Study 2014	Yes	Primary	**Multi-level regressions** and LLRs.	No	School attended		no strong evidence of clustering of self-harm, suicide ideation or suicide attempts in schools and school classes was found.
**Leung, Chow, Ip and Yip**	2018	Pure spatial and space-time clusters of self-harm in Kwai Tsing 2004-2012	Regional- Kwai Tsing, Hong Kong<br><br>Data obtained from the Hospital Authority hospital inpatient electronic database.and A&E database<br><br>2004-2012<br><br>9-year period	Yes	Primary	Spatial smoothing of the crude standardized mortality ratio (SMR) by Empirical Bayes method. **Pearson's chi-square** test and independent t -test to determine the significant difference between cases within the clusters and the rest of area.	Yes ArcGIS was used to perform spatial analysis. **SaTScan** was used to conduct space and space-time analysis.	Residency area	Yes	four significant high-risk pure spatial clusters of self-harm were identified<br><br>One signification space-time cluster was identified.
**Karami, Yazdi-Ravandi, Ghaleiha & Olfatifar**	2018	Comparison of the Clusters and Non-Clusters Areas of Attempted Suicide Cases in Hamadan Province, Western Iran: Findings from a Pilot Study (2016-2017)	City - Hamadan, western Iran<br><br>Data obtained from a suicide registry system in Sina Hospital, affiliated to Hamadan University of Medical Science<br><br>Oct 2016- Feb 2017<br><br>5-month period	No	Primary	**Logistic regression** analysis and **Pearson's chi-square** were used to more investigation of the clusters areas and compare them with other areas. **Montecarlo** simulation applied.	Yes<br><br>SaTScan applied.	Residency area	Yes	Two primary spatial clusters detected.

**Goodman, Puffer, Keiser & Gitari**	2020	Suicide clusters among young Kenyan men	County – Meru, Kenya<br><br>Data obtained from interviews with participants selected from 11 townships around Maua Methodist Hospital in Meru County, Kenya. based on characteristics reported at the hospital, where the majority of suicide attempters are young adult males.<br><br>532 interviews completed with young male adults aged 18-34.	No	Primary	Multiple logistic regression with survey-adjusted error terms was used to evaluate a trend between number of reported suicide attempts among friends and engaging in suicide ideation	No	social links	Yes	Number of clusters not reported.<br><br>there is a clear and significant trend between suicide ideation and number of friends who have attempted suicide. For each additional friend who attempted suicide, the odds of reporting suicidal ideation oneself increased by 90 percent. This indicates the presence of suicide clusters.

Table S4. Mass clusters

Authors	Year	Title	Level of data<br><br>Source(s) of data<br><br>Period of analysis	Aggregated data (Y/N)	Primary or secondary aim of the study	Type of analysis	Geospatial analysis (Y/N)	Clusters detected (Y/N)	Further details
**Jang, Sung, Park, Jeon**	**2016**	Copycat Suicide Induced by Entertainment Celebrity Suicides in South Korea.	National- South Korea.<br><br>Seven celebrity suicides were examined and defined using the Korean Integrated Newspaper Database System (KINDS).<br><br>4 periods of 28 days post celebrity suicide:<br><br>Micro data based on suicides obtained from Statistics Korea for period of 2005-2008<br><br>4-year period of observation	No	Primary	**Poisson time-series auto regression model** was used to estimate the relative risk of the total suicide number for each affected period. **Logistic regression** analysis was performed to investigate whether there were specific increases in the numbers of suicides in subgroups matching each celebrity.	No	Yes	Significant copycat effect induced by celebrity suicide was found, specifically in subgroups matching each celebrity in terms of sex, age and method.
**Jonas**	1992	Modelling and Suicide – A test of the Werther Effect	Regional- Baden-Wurttemberg, Germany. Suicide data sourced from the Statistics Office of Baden-Wurttemberg for years 1968-1980.	No	Primary	In line with previous research on the Werther Effect, a quasi-experimental method and a **time series regression analysis**. The frequency of suicides in an ‘experimental’ time period after the publication of the suicide was compared with the frequency in control periods.	No	Yes	Each of two different statistical methods (sign test, time series analysis) revealed an increase in suicide frequency after news about suicides of prominent persons.

**Chen, Tsai, Chen, Fan, Hung & Chen**	2010	Effect of media reporting of the suicide of a singer in Taiwan: the case of Ivy Li	Regional – Taipei, Taiwan. Sixty-three suicide attempters registered in a surveillance system of Taipei City Suicide Prevention Centre were assessed using a structured interview soon after media reporting of the suicide of a young female singing star.	No	Primary	Univariate and multivariate logistic **regression analyses** were conducted to compare differences in socio demographic (age, gender) and clinical characteristics between individuals who encountered with Li's news and those who did not. Both crude and adjusted odds ratio (OR) and 95% confidence interval (CI) were computed for individual predictors.	No	Yes	Forty-three (68%) respondents had encountered with the suicide news. Among them, 37% reported being influenced by the media reporting on their subsequent suicide attempts. There was a positive modelling effect in method of suicide (charcoal burning)
**Phillips, Carstensen**	1986	Clustering of teenage suicides after television news stories about suicide	National – United States of America. Daily fluctuations in a total of 12585 suicides in American teenagers before and after news or feature stories about suicide televised from 1973 to 1979.	Yes	Primary	**Regression analysis**	No	Yes	The more networks that carried a story about suicide, the greater the increase in suicides in the aftermath
**Queinec, Benjamin, Beitz, Lagarde, Encrenaz**	2010	Suicide contagion in France: an epidemiologic study	National – France. French daily data on suicides from 1979 to 2006 was obtained from the CepiDC (INSERM). Six most famous celebrities whose suicide occurred within this period were selected for analysis. Their name appeared at least 100 000 times in a popular web search engine.	Yes	Primary	Data were analysed using the **SARIMA model.**	No	Yes	Pierre Beregovoy (politician) and Kurt Cobain (musician) suicides were followed by a significant increase in the number of suicides in the French general population (+17.7% and +9.2% respectively). Dalida (singer) suicide had a significant effect among people aged from 45 to 59 only Sourire (singer) suicide among women only. No effect was found after Nino Ferrer (singer) and Gilles Deleuze (philosopher) suicides. Some of these suicides were strongly covered by the media; a qualitative analysis showed that, in most of cases, media did not follow the existing guidelines about suicide report.

**Cheng & Chen & Yip, Paul**	2011	The Foxconn suicides and their media prominence: is the Werther Effect applicable in China?	National – China. Relevant articles were collected from representative newspapers published in three cities in China: Beijing, Shenzhen, Guangzhou HK, and TW, together with searching intensity data on the topic conducted using the Baidu search engine in Mainland China.	Yes	Primary	The temporal clustering effects of the Foxconn suicides and their media prominence were assessed using **the Kolmogorov-Smirnov test**. The media reports of the Foxconn suicides' temporal patterns were explored using a nonparametric curve estimation method (that is, the local linear method). The potential mutual interactions between the Foxconn suicides and their media prominence were also examined, using **logistic and Poisson regression methods.**	No	Yes	The results support a temporal clustering effect for the Foxconn suicides. The occurrence of a Foxconn suicide also immediately influenced the intensity of both Baidu searching and newspaper reporting.
**Koburger, Mergl, Rummel-Kluge, Ibelshäuser, Meise, Postuvan, Roskar, Székely, Ditta Tóth, van der Feltz-Cornelis, Hegerl.**	2015	Celebrity suicide on the railway network: Can one case trigger international effects?	International -Data on railway suicides, overall suicide and from GoogleTrends for Germany, Austria, Hungary, the Netherlands and Slovenia were included. Those countries have been chosen due to their participation in the OSPI-Europe project.	No	Primary	**Mann-Whitney tests** were used to compare median daily numbers of railway suicides and the two-week period after the death of Robert Enke. A negative binomial regression analyses for count variables was selected to compute the incidence ratio of daily railway suicides within the two-week index period and the 8 week control period.	No	Yes	An increase of 15.5 railway suicides within the two weeks after the death of Robert Enke was significant in the four countries other than Germany.
**Ladwig, Kunrath, Lukaschek, Baumert**	2012	The railway suicide death of a famous German football player: impact on the subsequent frequency of railway suicide acts in Germany	National- Germany. German Railway Event database Safety (EDS) data	Yes	Primary	an inter-year approach comparing the incidence of RS during a predefined "index period" with identical time windows in 2006 to 2008; second, an intra-year approach comparing the number of RS 28 days before and after the incidence. Incidence ratios with 95% confidence intervals were estimated by **Poisson regression.**	No	Yes	The substantial increase of RS in the aftermath of the footballer's suicide death brought about copycat behaviour in an unforeseen amount, even though the media reporting was largely sensitive and preventive measures were taken.

**Etzersdorfer, Sonneck, Nagel-Kuess**	1992	Newspaper reports and suicide	Regional- Vienna, Austria. Suicide death stats for Vienna	No	Primary	**Mann-Whitney U test**	No	No	N/A. Link between decrease in reports and length of reports of suicide in Vienna subway and decrease in deaths reported after publication of media guidelines for reporting on suicides. This relation supports the hypothesis that media reports of suicide may trigger additional suicides.
**Fink, Santaella-Tenorio & Keyes**	2018	Increase in suicides the months after the death of Robin Williams in the US	National- USA<br><br>Monthly suicide count data in the US (1999±2015) were from the Centers for Disease Control and Prevention Wide-ranging ONline Data for Epidemiologic Research (CDC WONDER).	Yes	Primary	**Time-series analysis** was used to determine the expected number of suicides during the months following Williams' death. **SARIMA** model for this study.	No	Yes	a rapid increase in suicides in August 2014, and specifically suffocation suicides, that paralleled the time and method of Williams' death was found, as well as a dramatic increase in news media reports on suicides and Robin Williams during this same period, suggesting a connection between Williams' death and the subsequent increase in suicide deaths from August to December 2014.
**Whitley, Fink, Santaella-Tenorio, Keyes**	2019	Suicide Mortality in Canada after the Death of Robin Williams, in the Context of High-Fidelity to Suicide Reporting Guidelines in the Canadian Media	National- Canada<br><br>Data obtained deidentified monthly suicide count data from January 1999 to December 2015 from Statistics Canada.	Yes	Secondary	**time-series analyses** was used to estimate the expected number of suicides in the months following Robin Williams' death. This was done using a **SARIMA** method. Expected suicides were then compared with observed suicides	No	Yes	The time-series model indicated a 16% increase in the expected number of suicides during the months from August to December 2014 inclusive. Moreover, males over 30 had the greatest number of excess suicides, and suicides by suffocation (the method used by Robin Williams) were also higher in August and the following months.
**Sinyor, , Williams Tran, Schaffer, Kurdyak, Jane Pirkis & Niederkrotenthaler**	2019	Suicides in Young People in Ontario Following the Release of "13 Reasons Why	Regional – Ontario, Canada<br><br>data based on individuals 30 years and younger from January 2013 to March 2017	Yes	Primary	**Time-series analyses** were performed using data from January 2013 to March 2017 to predict expected deaths from April to December 2017 with a simple **seasonal model.**	No	Yes	Modelling predicted 224 suicides; however, 264 were observed corresponding to 40 more deaths or an 18% increase. In the primary analysis, monthly suicides exceeded the 95% confidence limit for 3 of the 9 months (May, July, and October).
**Niederkrotenthaler, Stack, Till, Sinyor, Pirkis, Rockett, Tran**	2019	Association of Increased Youth Suicides in the United States With the Release of 13 Reasons Why	National – USA.<br><br>Data obtained from the Centers for Disease Control and Prevention's WONDER Database<br><br>1999- 2017	Yes	Primary	Autoregressive integrated moving average **time series models** were fitted to the pre–April 2017 period to estimate suicides among the age groups and to identify changes in specific suicide methods used. The models were fitted to the full time series with	No	Yes	For 10- to 19-year-old males and females, increases in the observed values from April to June 2017 were outside the 95% confidence bands of forecasts. Models testing 3-month associated suicide mortality indicated 66 (95% CI, 16.3-115.7) excess suicides among males (12.4% increase; 95% CI, 3.1%-21.8%) and 37 (95% CI, 12.4-61.5) among females (21.7% increase; 95%

						dummy variables for (1) April 2017 and (2) April 1, 2017, to June 30, 2017.			CI, 7.3%-36.2%). The increase in the hanging suicide method was particularly high at 26.9%
**Lee**	2019	Media Coverage of Adolescent and Celebrity Suicides and Imitation Suicides among Adolescents	National – South Korea<br><br>Data obtained from Statistics Korea<br><br>Media reports obtained from news aggregator, Naver.com<br><br>2010-2015<br><br>16-year period	Yes	Primary	**A regression model** was constructed to test hypotheses 1 & 2. The model was estimated using the **Poisson estimation method.** A control variable was used to account for periods when no media coverage of suicide occurred. This was compared with 2-week windows after an adolescent or celebrity suicide was reported.	No	Yes	more suicides seem to occur among adolescents during the two weeks that follow the news media coverage of an adolescent's suicide. increase in the suicide rates among adolescents in the weeks following the news media coverage of a celebrity suicide varied according to the nationality of the celebrity, i.e. increase more significant when celebrity was of the same nationality as the individual.
**Pirkis, Currier, Too, Spittal**	2019	Suicides in Australia following media reports of the death of Robin Williams	National – Australia<br><br>Data obtained from the National Coroners Information System<br><br>2001-2016<br>16 year period	Yes	Primary	**Interrupted time series regression** to determine whether there were changes in suicides in the 5 month period immediately after Robin Williams' death	No	Yes	An 11% increase in suicide in the 5 month period following Williams' death, mainly accounted for by males aged 30-64 and by individuals who died by hanging (the method used by Williams)
**Sinyor. Tran, Garcia, Till, Voracek, Niederkrotenthaler**	2020	Suicide mortality in the United States following the suicides of Kate Spade and Anthony Bourdain	National – USA<br><br>Data obtained from the USA Centers for Disease Control and Prevention's public-use mortality file<br><br>1999-2018<br>20 year period	Yes	Primary	**time-series analysis was performed**, examining monthly suicide data by age group (⩽19, 20-44, 45-64 and ⩾65 years), for both men and women, for all suicide methods and for hanging versus non-hanging methods. **SARIMA models** were fitted to the pre-June 2018 period, estimating suicides in subsequent months and identifying deviations from expected values.	No	Yes	There were 418 more suicides than expected, including 275 excess suicides in men and 182 (in women. These equate to 4.8%, 4.1% and 9.1% increases above expected counts.
**Sinyor, Williams, Schaffar, Pirkis, Niederkrotenthaler**	2019	Suicides in Young People in Ontario Following the Release of "13 Reasons Why"	Province- Ontario, Canada<br><br>Data obtained from the Office of the Chief Coroner<br><br>2013-2017<br>4 year period	No	Primary	**Time-series analysis, involving a simple seasonal model. The ARIMA model** was further applied to estimate the possible effect of 13RW in the period April to December 2017	No	Yes	Modeling predicted 224 suicides, however 264 were observed corresponding to 40 more deaths or an 18% increase. In the primary analysis, monthly suicides exceeded the 95% confidence limit for 3 to 9 months.

**Bridge, Greenhouse, Ruch, Horowitz, Kelleher, Campo**	2019	Association Between the Release of Netflix's 13 Reasons Why and Suicide Rates in the United States: An Interrupted Time Series Analysis	National- USA<br><br>Data obtained from US national monthly suicide rates<br><br>2013-2017<br>4 year period	Yes	Primary	**1. an interrupted time series segmented regression** accounting for both the underlying secular trend & seasonal variation in youth suicide rates 2. **the Holt-Winters' forecasting method,** a triple exponential smoothing model to fit and forecast monthly suicide rate data: one equation for level, one for secular trend, and one for seasonality.	No	Yes	The release of 13 Reasons Why was associated with a significant increase in monthly suicide rates among US youth aged 10 to 17 years.
**Romer**	2020	Reanalysis of the Bridge et al. Study of suicide following release of 13 reasons why	National- USA<br><br>Information detailing source of data not provided<br><br>2013-2017<br>4 year period	Yes	Primary	**Auto-regression analysis** that accounted for long-term time trend, autocorrelation, and a discontinuity in the trend following the release of the first season of the show. After removing auto-correlation, the resulting series were tested using **OLS regression** to identify changes in suicide rates for the month of April 2017, the month the show first appeared, and for three months following the release of the show.	No	No	a small but nonsignificant increase in suicide in April that was unique to that month, potentially consistent with a combined protective and harmful effect of the show.

Table S5. Echo clusters

Authors	Year	Title	Level of suicide mortality data<br><br>Source of data<br><br>Period of analysis	Aggregated data (Y/N)	Primary or secondary aim of study	Type of analysis	Geospatial (Y/N)	Level of geographical data	Clusters detected (Y/N)	Number of clusters detected
**Too, Pirkis, Milner, Robinson, Spittal**	2018	Clusters of Suicidal Events among Young People: Do Clusters from one time period predict later clusters?	Regional – New South Wales, Australia.<br><br>Data obtained from the Australian Bureau of Statistics<br><br>NSW 2001-2012<br><br>13-year period<br><br>Western Australia 2000-2011<br><br>12-year period	No – individual level data.<br><br>Suicide and Suicide Attempts amalgamated and categorised in one variable – suicidal events	Primary	**Poisson discrete** scan statistic.<br>We used **Monte Carlo** simulation to assess the significance level of any potential clustered that we detected (Kulldorff, 1997). Clusters were included if their p value was lower than .10, signifying the presence of a possible cluster.	Yes<br><br>Maps were created using **ArcGIS**.<br><br>SaTScan employed.	Residential address	Yes	In the analyses with time window of 1–12 months, we consistently identified 10 clusters in period one (10.2%, 1,439/14,049 events) and five clusters (5.9%, 777/13,174 events) in period two in NSW while identified six clusters in period one (12.7%, 393/3,095 events) and four clusters (2.9%, 191/6,552 events) in period two in WA.<br><br>It was found that earlier clusters predicted 36% of the later clusters occurring in the same areas.
